# Association between laboratory markers and Covid-19 disease severity and outcome: a retrospective cohort study in Saudi Arabia

**DOI:** 10.3389/fimmu.2023.1198530

**Published:** 2023-07-11

**Authors:** Aliaa Amr Alamoudi, Sahar Eldakhakhny, Haneen Banjar, Ghada Ajabnoor, Sama Badr Aljohani, Rasha Ramadan Basheer, Basmah Eldakhakhny, Mazen Badawi, Ayman Elsamanoudy

**Affiliations:** ^1^ Department of Clinical Biochemistry, Faculty of Medicine, King Abdulaziz University, Jeddah, Saudi Arabia; ^2^ Regenerative Medicine Unit, King Fahad Medical Research Center, King Abdulaziz Univeristy, Jeddah, Saudi Arabia; ^3^ Diagnostic Virology, King Abdulaziz University Hospital, King Abdulaziz University, Jeddah, Saudi Arabia; ^4^ Computer Science Department, Faculty of Computing and Information Technology, King Abdulaziz University, Jeddah, Saudi Arabia; ^5^ Center for Artificial Intelligence in Precision Medicine, King Abdulaziz University, Jeddah, Saudi Arabia; ^6^ King Abdulaziz and his Companions Foundation for Giftedness and Creativity “Mawhiba”, King Abdulaziz University, Jeddah, Saudi Arabia; ^7^ Restorative Dentistry Department, Faculty of Dentistry, King Abdulaziz University, Jeddah, Saudi Arabia; ^8^ Conservative Dentistry Department, Faculty of Dentistry, October University for Modern Sciences and Arts University, Cairo, Egypt; ^9^ Department of Medicine, Faculty of Medicine, King Abdulaziz University, Jeddah, Saudi Arabia; ^10^ Department of Medicine, King Faisal Specialist Hospital and Research Center, Jeddah, Saudi Arabia; ^11^ Medical Biochemistry and Molecular Biology, Faculty of Medicine, Mansoura University, Mansoura, Egypt

**Keywords:** laboratory parameters, Covid-19, disease severity, outcome, King Abdulaziz University Hospital (KAUH), albumin

## Abstract

**Introduction:**

In Saudi Arabia, limited studies have evaluated factors including epidemiologic, clinical, and laboratory findings that are associated with COVID-19 disease. The aim of this paper was to identify laboratory parameters used in King Abdulaziz University Hospital which show an association with disease severity and patient outcome in the form of mortality.

**Methods:**

Age, gender, medical history, and laboratory parameters were all retrospectively assessed concerning disease severity and disease outcome in a total of 111 COVID-19 patients at King Abdulaziz University Hospital between July 2020 and August 2020. Patients were categorized into mild disease if they did not require ward admission, moderate if they met the Ministry of Health criteria for isolation ward admition, and severe if they were admitted to the ICU.

**Results:**

Age but not gender was associated with the disease severity *X^2^* (4, N = 110) = 27.2, p <0.001. Of all laboratory parameters on admission, only the levels of Albumin appeared to be significantly associated *X^2^* (2, N =70) = 6.6, p <0.05 with disease severity. Age but not gender was also significantly associated with disease outcome *X^2^* (2, N = 110) = 12.8, p < 0.01. Interestingly, RBC count also showed a significant relation with disease outcome *X^2^* (2, N = 71) = 6.1, p <0.05.

**Discussion:**

This study provides more understanding of the laboratory characteristics in our part of the world to efficiently manage the disease.

## Introduction

In 2020, Corona Virus disease (COVID-19) was declared as a global pandemic by the World Health Organization (WHO) (1, 2). Up to October 2022, WHO reported a global total number of 623,000,396 confirmed cases including 6,550,033 deaths by the disease (3). Saudi Arabia was no exception, and like many other countries has undergone serious health threats with the pandemic. Up to October 2022, there have been 819,323 confirmed cases of COVID-19 with 9,384 deaths in Saudi Arabia reported to WHO (3). By October 2022, the 7-day average number of new cases was 191 (3). Such numbers confirm WHO’s perspectives that “…the COVID-19 pandemic remains an acute global emergency” and the risk for new variants and future surges remains a real threat.

Since the beginning of the pandemic, the disease exhibited a very variable presentation following an unforeseeable course, making it hard to predict disease outcomes in individuals (4, 5). A large number of infected patients were asymptomatic, while others showed a wide spectrum of symptoms and signs ranging from fever, cough, fatigue, shortness of breath in mild illness, to severe life threatening pneumonia and critical disease in the form of respiratory failure and multi- organ dysfunction. The disease is characterized by cytokine storm and coagulation abnormalities which ultimately can result in tissue destruction and vital organs dysfunction (4).

Similar to many countries world-wide, the pandemic exhibited a huge strain on Saudi Arabia’s healthcare system. A study conducted to assess hospital and ICU bed surge capacity during the pandemic concluded that there was an urgent need for hospital and ICU beds to accommodate critical cases in Saudi Arabia (6). Decisions thus related to postponing admission and discharging patients from wards are crucial and require critical assessment in addition to reliable predictors that can predict disease severity (6).

Previous studies have investigated the relationship of blood/serum biomarkers with disease severity and disease outcomes. High neutrophil count, lymphopenia, high C-reactive protein (CRP), Lactate dehydrogenase (LDH), Ferritin, and IL-6, for example, were all shown to be predictors of disease severity in various studies world-wide. In Saudi Arabia, however, very limited studies have evaluated effective indicators which correlated with the disease including epidemiologic, clinical, and laboratory findings. In a retrospective study conducted at King Abdulla Hospital in Bisha province between March 20 and June 30 2022, a high LDL level and neutrophil count and low lymphocyte count was found in all patients (7). In addition, a significant association was seen between cardiovascular diseases, hypertension, renal failure, old age, and death from the disease. In another study, conducted in four private tertiary hospitals and one private hospital in Riyadh, aspartate aminotransferase (AST), CRP, D-dimer, and ferritin were all significantly higher in moderate cases in comparison to mild cases (8). It is worth noting however that those factors were assessed during the first three months of the pandemic which showed special circumstances in terms of mandating hospital isolation, admission criteria, and management plans.

King Abdulaziz University Hospital (KAUH) in Jeddah is one of the largest educational hospitals in Saudi Arabia with a very multi-cultural patient population and played an essential role in accommodating COVID-19 cases. The aim of this paper was to define the laboratory markers used in KAUH that showed an association with disease severity, and patient outcome in the form of mortality during the period between July 2020 and August 2020.

## Materials and methods

### Study population and data collection

This study was a retrospective study conducted at KAUH in Jeddah, Saudi Arabia and approved by the Biomedical Research Ethics Committee ethics board at King Abdul-Aziz University (Reference No 360-20).

The study included all COVID-19 patients, confirmed by nasopharyngeal swab RT-PCR for SARS-CoV-2, admitted between July 2020 and August 2020, which was a total of 151 patients. Excluded were pediatric patients, pregnant women, and those who did not have any electronic medical records, leading to a total inclusion of 111 patients. Patients were categorized into Mild, Moderate and Severe disease. Mild disease if they did not require ward admission, which was equivalent to categories 1 and 2 according to the WHO ordinal clinical severity scale i.e ambulatory without activity limitation or with activity limitation. Moderate if they were admitted to the isolation ward which was equivalent to categories 3,4 and 5 on the WHO scale. Severe if they were admitted to the ICU which was equivalent to categories 6,7 and 8. Confirmed cases were admitted to the isolation ward if they met the Ministry of Health SCDC guideline criteria, which included patients who were symptomatic and showed signs of severe disease such as low oxygen saturation SpO2<94% on room air, hemodynamic instability, clinical or radiographic evidence of pneumonia. In addition, patients who were at risk of severe disease such as co-morbidities and immunocompromised patients, especially if they showed signs of active cytokine release, were admitted. Patients who showed deterioration clinically and were not able to main oxygen saturation with non-invasive methods of oxygenation were admitted to ICU.

A standard laboratory workup was done for all confirmed COVID-19 patients which ensured that all routine laboratory tests were requested for all patients and at standardized time points during their admission.

A predesigned form was designed to collect data from patients’ electronic medical records. Data included age, gender, past medical history, length of hospital stay, patient outcome which was defined as recovery if patients was discharged or death in cases of in-hospital mortality, COVID-19 complications which included new events (unrelated to previous medical history) of pneumonia, organ failure, cardiac problems, blood clots and acute kidney injury, in addition to all the laboratory parameters ordered for patients.

### Statistical analysis

Data analysis was conducted using SPSS version 25. Descriptive data were described as frequency (N) and percentage. Shapiro-Wilk test was used to check if continuous variables follow a normal distribution. Mann-Whitney U test and Kruskal-Wallis test were used when comparing two or more than two groups, respectively. For further analysis and to facilitate application of statistical and machine learning models in future work, continuous data was transferred to categorical data (low, normal, high). The chi-squared test for independence was used to test the association between the categories. Dependent study variables were defined as a binary outcome. A Binary Logistic Regression Model (BLRM), with Enter Criteria=0.05 and Elimination=0.10 was used to determine the significant predictors of any given dependent study variables with 95% confidence intervals. A General Linear Model (GLM) univariate analysis was also used to identify significant predictors using Main Effect as model. A P-value less than 0.05 was considered statistically significant.

## Results

Descriptive data of COVID-19 cases is presented in **Table 1**. Overall, the majority of cases were mild cases that required only an ER visit without admission (41.4%), while 39.6% of cases were moderate which were ward-admitted, and 18.9% were severe cases that required ICU admission. 88.3% of the cases recovered during their hospital stay and were discharged in stable condition, while the remaining 11.7% were inpatient mortality cases. Hospital stay was less than 7 days in 65.7% of cases, and the majority of patients (70.3%) did not develop any COVID-19-related complication.

**Table 1:** Descriptive data of COVID-19 cases.

	Frequency(N)	Percent (%)
Severity		
Mild	46	41.4
Moderate	44	39.6
Severe	21	18.9
Total	111	100.0
Complication		
No complication	78	70.3
Complication	33	29.7
Total	111	100.0
Outcomes		
Recovery	98	88.3
Inpatient mortality	13	11.7
Total	111	100.0
Hospital stay		
< 7 days	73	65.7
≥ 7 days	38	34.2
Total	111	100.0
History of Medical disease		
No medical condition	63	56.8
Medical condition	48	43.2
Total	111	100.0
Gender		
Male	67	60.4
Female	44	39.6
Age		
18-44	50	45.0
45-64	42	37.8
≥ 65	18	16.2

In terms of the laboratory parameters measured upon admission (including ER admission), most patients fell in the normal range of most parameters except for lymphocyte, monocyte and eosinophil count, D-dimer, ferritin, Albumin, AST, and LDH levels (**Table 2**). 86.3%,45.1%, and 82.2% of all patients showed low lymphocyte, monocyte and eosinophil count, respectively. 62.7% of patients showed high D-dimer levels and 74.1% had high ferritin levels. Albumin levels were low in 68.6% of patients, while AST and LDH levels were high in 57.1% and 84.7% of patients respectively.

**Table 2:** Laboratory parameter ranges in admitted patients.

Parameter		Frequency (N)	Percent (%)
Hgb	Low	27	36.9
	Normal	44	60.3
	High	2	2.7
	Total	73	100.0
RBC count	Low	34	46.6
	Normal	36	49.3
	High	3	4.1
	Total	73	100.0
PLT	Low	13	17.8
	Normal	56	76.7
	High	4	5.5
	Total	73	100.0
WBC	Low	18	24.6
	Normal	45	61.6
	High	10	13.8
	Total	73	100
Neutrophils	Low	11	15.1
	Normal	45	61.6
	High	17	23.3
	Total	73	100.0
*Lymphocytes	Low	63	**86.3**
	Normal	10	13.7
	Total	73	100
*Monocytes	Low	32	**45.1**
	Normal	30	42.3
	High	9	12.6
	Total	71	100
*Eosinophils	Low	60	**82.2**
	Normal	10	13.7
	High	3	4.1
	Total	73	100
Basophils	Low	8	11
	Normal	60	82.2
	High	5	6.8
	Total	73	100
*D-Dimer	Normal	22	37.3
	High	37	**62.7**
	Total	59	100
PT	Normal	50	79.4
	High	13	20.6
	Total	63	100
APTT	Normal	57	92
	High	5	8
	Total	62	100
INR	Normal	50	79.4
	High	13	20.6
	Total	63	100
*Ferritin	Normal	15	25.9
	High	43	**74.1**
	Total	58	100
Na	Low	33	45.2
	Normal	40	54.8
	Total	73	100
K	Low	13	17.8
	Normal	53	72.6
	High	7	9.6
	Total	73	100
Cl	Low	9	12.3
	Normal	64	87.7
	Total	73	100
Urea	Low	7	9.6
	Normal	42	57.5
	High	24	32.9
	Total	73	100
Creatinine	Low	12	16.7
	Normal	43	59.7
	High	17	23.6
	Total	72	100
Total protein	Low	4	5.7
	Normal	59	84.3
	High	7	10
	Total	70	100
*Albumin	Low	48	**68.6**
	Normal	22	31.4
	Total	70	100
ALP	Normal	65	91.5
	High	6	8.5
	Total	71	100
*AST	Low	3	4.3
	Normal	27	38.6
	High	40	**57.1**
	Total	70	100
ALT	Normal	54	76.1
	High	17	23.9
	Total	71	100
GTT	Normal	47	66.2
	High	24	33.8
	Total	71	100
Total Bilirubin	Normal	64	90.1
	High	7	9.9
	Total	71	100
*LDH	Normal	9	15.3
	High	50	**84.7**
	Total	59	100

*Indicate paramters in which the majority of patients (bold) fell either higher or lower than normal range.

**Figure 1:**
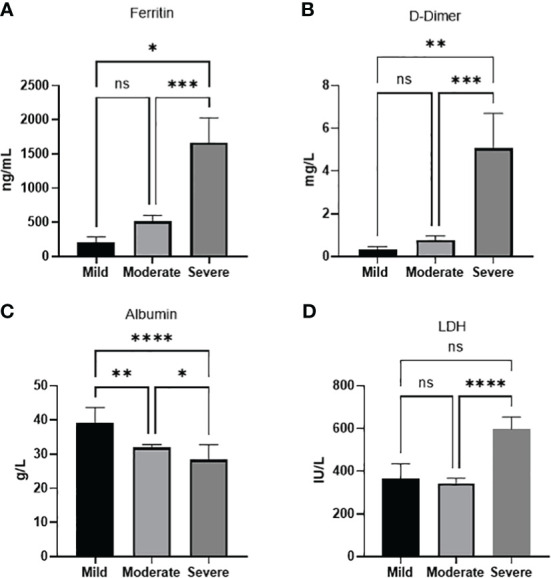
Laboratory paramaters associated with disease severity. **(A)** Ferritin **(B)** D-dimer, **(D)** LDH levels increase with disease severity. **(C)** Albumin levels decreases with disease severity. Data presented as mean +/- SEM. * p value <0.05,** p <0.01,*** p <0.001,**** p <0.0001, ns indicates not significant (p>0.05).

In order to study the relationship between the various parameters and COVID-19 severity, a chi-square test of independence was performed to look for significant association between each category and disease severity (**Table 3**). Developing a COVID-19-related complication, and disease outcome (recovery or inpatient mortality) was, as expected, associated with disease severity *X^2^* (2, *N* = 111) = 69.2, *p <*0.001 and *X^2^* (2, *N* = 111) = 63.1, *p <*0.001, respectively. History of a previous medical condition was also associated with disease severity *X^2^* (2, *N* = 111) = 48.7, *p <*0.001. Age but not gender was associated with the disease severity *X^2^* (4, *N* = 110) = 27.2, *p <*0.001. There was a significant difference between patients ≥65 and patients between 45-64 compared to patients between 18-44 (*p*<0.0001, *p*<0.001 respectively) in terms of disease severity, with the former groups showing more severe disease. However, no significant difference in disease severity was found between patients ≥65 and patients between 45-64. Interestingly, out of all the laboratory parameters on admission, only the levels of Albumin appeared to be significantly associated *X^2^* (2, *N* =70) = 6.6, *p* =0.036 with disease severity, however, it is worth mentioning that ALT levels reached a p-value of 0.059, *X^2^* (2, *N* =71)=5.6. To further confirm the association between the parameters and disease severity an ANOVA analysis was done for each of the significant parameters. We found that severe cases showed a significantly higher level of ferritin when compared to moderate *(p*<0.001) or mild cases (*p*<0.05). D-dimer was also significantly higher in severe cases compared to moderate (*p*<0.001) or mild cases (*p*<0.01). LDH severe cases showed a significantly higher level compared to moderate cases (*p*<0.0001). Intriguinly, Albumin levels were not only significantly lower in severe cases compared to moderate disease (*p*<0.05) and mild cases (*p*<0.0001), but was also significantly lower in moderate disease compared to mild (*p*<0.01) (**Figure 1**).

**Table 3:** Demographic and laboratory parameters associated with COVID-19 severity.

		Mild (N)	Moderate (N)	Severe (N)	N, X^2^,df	P
Complication	No complication	46	32	0	111,69.2,2	**<.001**
	Complication	0	12	21		
Outcome	Recovery	46	44	8	111,63.1,2	**<.001**
	In-patient mortality	0	0	13		
Hx of Medical disease	No medical condition	44	14	5	111,48.7,2	**<.001**
	Medical condition	2	30	16		
Gender	Male	25	25	17	111,4.6,2	.098
	Female	21	19	4		
Age	18-44	32	16	2	110,27.2,4	**<.001**
	45-64	11	20	11		
	≥ 65	2	8	8		
Hgb	Low	2	15	10	73,5.8,4	.214
	Normal	6	28	10		
	High	1	0	1		
RBC count	Low	4	20	10	73,8.2,4	.084
	Normal	5	23	8		
	High	0	0	3		
PLT	Low	0	7	6	73,5.4,4	.243
	Normal	9	34	13		
	High	0	2	2		
WBC	Low	2	13	3	73,4.8,4	.300
	Normal	7	25	13		
	High	0	5	5		
Neutrophils	Low	0	6	5	73,5.0,4	.283
	Normal	8	27	10		
	High	1	10	6		
Lymphocytes	Low	9	36	18	73,1.6,2	.432
	Normal	0	7	3		
Monocytes	Low	7	17	8	71,4.9,4	.292
	Normal	1	20	9		
	High	1	5	3		
Eosinophils	Low	7	34	19	73,2.5,4	.630
	Normal	2	7	1		
	High	0	2	1		
Basophils	Low	3	4	1	73,8.2,4	.084
	Normal	5	35	20		
	High	1	4	0		
D-Dimer	Normal	5	12	5	59,2.5,2	.278
	High	3	23	11		
PT	Normal	5	28	17	63,3.9,2	.141
	High	1	11	1		
APTT	Normal	5	35	17	62,0.7,2	.686
	High	1	3	1		
INR	Normal	6	28	16	63,3.9,2	.141
	High	0	11	2		
Ferritin	Normal	2	10	3	58,0.4,2	.816
	High	6	25	12		
Na	Low	3	19	11	73,0.9,2	.617
	Normal	6	24	10		
K	Low	1	9	3	73,7.6,4	.104
	Normal	8	32	13		
	High	0	2	5		
Cl	Low	1	3	5	73,3.7,2	.156
	Normal	8	40	16		
Urea	Low	2	5	0	73,6.7,4	.152
	Normal	6	25	11		
	High	1	13	10		
Creatinine	Low	2	7	3	72,3.3,4	.504
	Normal	7	25	11		
	High	0	11	6		
Total protein	Low	0	2	2	70,2.3,4	.681
	Normal	8	36	15		
	High	1	3	3		
Albumin	Low	4	27	17	70,6.6,2	**<.036**
	Normal	5	15	2		
ALP	Normal	8	39	18	71,0.2,2	.888
	High	1	3	2		
AST	Low	0	3	0	70,2.7,4	.601
	Normal	3	15	9		
	High	6	24	10		
ALT	Normal	4	34	16	71,5.6,2	.059
	High	5	8	4		
GTT	Normal	4	30	13	71,2.4,2	.297
	High	5	12	7		
Total Bilirubin	Normal	8	37	19	71,0.7,2	.689
	High	1	5	1		
LDH	Normal	1	5	3	59,0.1,2	.937
	High	7	29	14		

Bold values indicate significant values.

Given that age and low albumin levels were two statistically significant categorical parameters associated with disease severity we wanted to study which would be more related to disease severity. Using GLM suggested that between the two significant factors age better predicts disease severity compared to albumin levels (**Table 4**). Age group 18-44 were expected to have a decrease in disease severity with a -0.419 [-0.821, -0.017] with a *p*=0.041.

**Table 4:** Variables and Disease severity.

Parameter	B	S.E.	95% Confidence Interval		p-value
			Lower Bound	Upper Bound	
Age = 18-44	-0.419	0.201	-0.821	-0.017	0.041^a^
Age = 45-64	-0.057	0.180	-0.417	0.303	0.752
Albumin = Low	0.279	0.158	-0.037	0.595	0.083

a-significant using General Linear Model at <0.05 level.

B= Logistic regression coefficient

S.E.= Standard error

**Figure 2:**
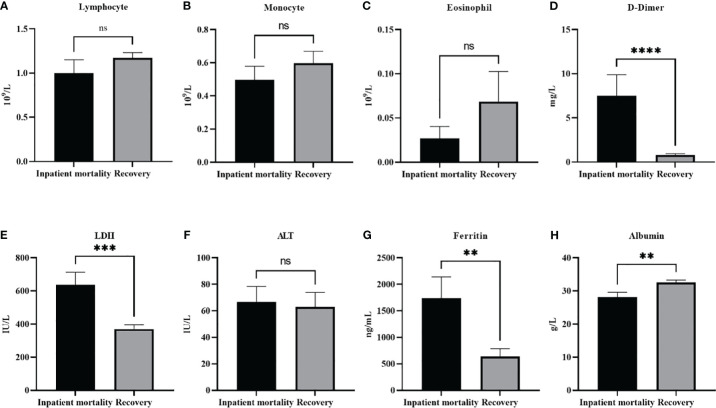
Laboratory paramters association with patients’ outcomes. The level of different white blood cells did not change with patients’ outcomes **(A)**. lymphocyte, **(B)** moncyte **(C)** eosinophil. On the other hand **(D)** D-dimer, **(E)** LDH, and **(G)** Ferrtin levels were all significantly higher in in patient mortality. **(F)** ALT levels were not effected by patients’ ouctome, while **(H)** Albumin levels were significantly decreased in patient mortality. Data presented as mean +/- SEM. ** p <0.01,*** p <0.001,**** p <0.0001, ns indicates not significant (p>0.05).

**Table 5** demonstrates the analysis of the various demographic and laboratory parameters categories in association with the disease outcome (inpatient mortality or recovery). As expected, there was a significant relationship between Covid complications and disease outcome *X^2^* (1, *N* = 111) = 34.8, *p <*0.001. In addition, a history of previous medical conditions were associated with disease outcome *X^2^* (1, *N* = 111) = 4.0, *p* =0.044. Although gender was not associated, age was significantly associated with disease outcome *X^2^* (2, *N* = 110) = 12.8, *p <*0.01. Patients ≥ 65 and patients between 45-64 showed a significantly higher inpatients mortality compared to patients between 18-44 (*p*<0.001, *p*=0.026 respectively). No significant difference was seen though between patients ≥ 65 and patients between 45-64. Compellingly, out of all the measured laboratory parameters, only RBC count showed a significant relation with disease outcome *X^2^* (2, *N* = 71) = 6.1, *p* =0.046. With further analysis of the continuous variables, we found that the same factors that were associated with disease severity were also associated with increased mortality (**Figure 2**). Significantly higher levels of LDH (*p*<0.001), D-dimer (*p*<0.0001), and ferritin (*p*<0.01), were found in in-patient mortality, while a significantly decreased level of Albumin (*p*<0.01) was seen in in-patient mortality. Other factors such as ALT or different types of white blood cells were not associated with patient recovery or mortality.

**Table 5:** Demographic and laboratory parameters associated with COVID-19 outcome.

		Inpatient mortality	Recovery	N, X2, df	P-value
Complication	No complication	0	78	111,34.8,1	**<.001**
	Complication	13	20		
Hx of Medical disease	No medical condition	4	59	111,4.0,1	**<.044**
	Medical condition	9	39		
Gender	Male	9	58	111,0.4,1	.487
	Female	4	40		
Age	18-44	1	49	110,12.8,2	**<.002**
	45-64	6	36		
	≥ 65	6	12		
Hgb	Low	6	21	73,2.2,2	.317
	Normal	6	38		
	High	1	1		
RBC count	Low	7	27	71,6.1,2	<.046
	Normal	4	32		
	High	2	1		
PLT	Low	1	12	73,1.1,2	.555
	Normal	11	45		
	High	1	3		
WBC	Low	2	16	73,4,2	.131
	Normal	7	38		
	High	4	6		
Neutrophils	Low	1	10	73,0.9,2	.621
	Normal	8	37		
	High	4	13		
Lymphocytes	Low	11	52	73,0.0,1	.845
	Normal	2	8		
Monocytes	Low	5	27	71,0.9,2	.615
	Normal	7	23		
	High	1	8		
Eosinophils	Low	12	48	73,2.8,2	.240
	Normal	0	10		
	High	1	2		
Basophils	Low	1	7	73,1.4,2	.488
	Normal	12	48		
	High	0	5		
D-Dimer	Normal	3	19	59,0.5,1	.446
	High	8	29		
PT	Normal	11	39	63,3.4,1	.063
	High	0	13		
APTT	Normal	11	46	62,1.1,1	.279
	High	0	5		
INR	Normal	11	39	63,3.4,1	.063
	High	0	13		
Ferritin	Normal	2	13	58,0.0,1	.786
	High	7	36		
Na	Low	4	29	73,1.3,1	.249
	Normal	9	31		
K	Low	2	11	73,0.6,2	.729
	Normal	9	44		
	High	2	5		
Cl	Low	1	8	73,0.3,1	.575
	Normal	12	52		
Urea	Low	0	7	73,2.4,2	.301
	Normal	7	35		
	High	6	18		
Creatinine	Low	3	9	72,1.9,2	.375
	Normal	5	38		
	High	4	13		
Total protein	Low	1	3	70,0.2,2	.681
	Normal	10	49		
	High	1	6		
Albumin	Low	10	38	70,1.4,1	.226
	Normal	2	20		
ALP	Normal	11	54	71,0.0,1	.987
	High	1	5		
AST	Low	0	3	70,0.6,2	.719
	Normal	5	22		
	High	7	33		
ALT	Normal	9	45	71,0.0,1	.925
	High	3	14		
GTT	Normal	9	38	71,0.5,1	.479
	High	3	21		
Total Bilirubin	Normal	11	53	71,0.0,1	.846
	High	1	6		
LDH	Normal	1	8	59,0.2,1	.612
	High	9	41		

Bold values indicate significant values.

Using BLRM we found that when using age, disease severity, RBC count as variables, disease severity was the top predictor of disease outcome, followed by age, and RBC count (**Table 6**).

**Table 6:** Variables and Disease outcome.

Variables in the Equation	B	S.E.	Exp(B)	95% C.I.for Exp(B)		p-value
				Lower	Upper	
Age						0.719
Age(18-44)	0.956	1.667	2.601	0.099	68.292	0.566
Age(45-64)	0.791	1.049	2.206	0.282	17.227	0.451
RBC count						0.785
RBC(Low)	-0.048	1.453	0.953	0.055	16.428	0.974
RBC(Normal)	0.628	1.447	1.873	0.110	31.956	0.665
Disease severity						>0.999
Disease severity(Mild)	21.437	13155.737	2.042E+09	0.000		0.999
Disease severity(Moderate)	21.564	6017.472	2.317E+09	0.000		0.997
Constant	-1.246	1.460	0.288			0.394

## Discussion

This study reflected on the experience of one of the largest educational hospitals in Saudi Arabia, KAUH, to define the laboratory markers used and associated with COVID-19 severity and outcome. In our study, age, low albumin levels, high ferritin, D-dimer and LDH were significantly associated with disease severity and outcome. In addition, normal RBC count appeared as an important parameter for patient’s outcome.

In our study, 62.7% of all patients showed high levels of D-dimer. D-dimer has beenassociated with COVID-19 and the disease severity (9–11). In a study of 248 COVID patients in Wuhuan, D-dimer was elevated in 74.6% of patients (11). Levels also increased with disease severity (10, 11) and higher levels on admission were found to predict mortality in a few studies (10). In a Saudi study, higher D-dimer levels were also found in mild to moderate disease (8). D-dimer is a degradation product of fibrin which is regularly used for diagnosis of thrombotic disorders. Various pathological and non-pathological conditions are expected to increase fibrin production and breakdown, including venous thromboembolism, disseminated intravascular coagulation, liver diseases, pregnancy, and inflammatory process. However, infection has been one of the most frequent reasons behind elevated D-dimer in admitted patients (12). It is thought that both the acute lung injury and the thromboemobolitic dysregulation seen in COVID-19 are the cause of the elevated D-dimer (9).

The majority of patients in our study (74%) showed high levels of serum ferritin. Levels were also significantly higher in severe disease and in patient mortality. Serum ferritin is known as an acute phase reactant which acts as an important inflammatory marker. Hyperferrtinemia has been demonstrated in a range of diseases including rheumatoid disorders, cancer and others, indicating the activation of the macrophage-monocyte system. Given the systemic inflammatory feature of COVID-19, hyperferrtinemia has been associated with the disease severity in several studies including Saudi Arabia (8, 13, 14). In two metanalysis studies, high serum ferritin was significantly increased in severe patients compared with non-severe patients, and in non-survivors compared with survivors (13, 14). In addition, patients who required ICU admission had higher levels than those who did not require ICU or mechanical ventilation (14). However, it is worth noting that a high heterogeneity was seen between studies, which according to authors could be contributed to differences in mean age and concomitant co-morbidities seen in patients, both of which are expected to effect serum ferritin levels (14).

LDH was also associated with disease severity and in inpatient mortality. LDH is an intracellular enzyme which is mostly active in the lungs, heart, liver and skeletal muscles. Increased blood concentration is seen in tissue damage and cell death and is though to be an important marker for lung damage (15, 16). Several studies and meta-analysis studies have shown that the enzyme was associated with disease severity and was found in higher levels in ICU patients and non-survival patients (15, 16). In severe cases, LDH together with ferritin were found to be prognostic for ARDS development (17).

In this study, low albumin levels were found in the majority of patients. In addition, severe cases and patient morality showed significantly lower levels. Noteworthy, as a category, low albumin levels appeared to be the only laboratory parameter that was significantly associated with disease severity. Low serum albumin has been associated with disease severity in other studies (18–20). In a meta analysis study analyzing sixty-seven studies with a total of 19,760 COVID-19 patients, it was concluded that low albumin concentration were significantly lower in patients with severe disease (18). In addition, albumin was shown to be an independent risk for mortality (21, 22). It is known that albumin is a crucial protein that exerts homeostatic functions, and is the most essential protein for inducing oncotic pressure in the vascular system. Various reasons have been suggested to explain the hypoalbuminemia seen in severe cases of Covid-19. For example, impaired liver synthesis, or albumin loss by the kidney can be expected in severe cases, yet, this is not usually the case and indeed this was not reflected in the liver and kidney function biomarkers in our study. Reason explaining the low albumin concentration could be related to the characteristic inflammatory state of COVID-19, which due to vascular permeability could result in the extravasation of serum albumin into the interstitial fluid (18). Even so, it is also important to recognize that albumin concentrations tend to decrease with age, and given that age is associated with COVID disease severity, the relationship with albumin could be a secondary association. Emphasizing the importance of albumin, a recent retrospective study on 114 COVID-19 patients have demonstrated that albumin infusion to ICU patients with hypoalbuminemia was associated with shorter hospitalization, prolonged survival, and enhancement in some of the laboratory markers (23).

Interestingly in this study, normal and low levels of RBCs were associated with disease recovery. RBCs are responsible for oxygen transport from lungs to tissue. Despite this, their role as passive carriers has been challenged in the last decades (24, 25). Their processing and delivery of vasoactive factors such as NO is thought to result in vasoregulation that can caliber blood vessels and therefore regulate blood flow based on oxygen availability in the lung and consumption in the periphery (24). Another active role for RBC was further demonstrated when abnormalities in RBCs numbers and function was found to be associated with both arterial and venous thrombosis (25). For example, the risk of CVD is found to be two-fold greater in high hematocrit patients vs low hematocrit, in addition 20% of polycythemia patients present with thrombosis as their main symptom (25). This could be explained by RBCs being a primary factor in blood viscosity, and it is known that an increased blood viscosity is a main risk factor for thrombosis. A normal to low RBC count perhaps acts as a protective factor from developing a thrombotic event in COVID-19 patients, which might explain why normal and low RBC counts were associated with recovery from disease in our study. However, further larger studies would be required to investigate the impact of RBC counts and function in COVID-19 severity.

RBC are constantly exposed to endogenous and exogenous reactive oxygen species which require an anti-oxidant system to neutralize such excess ROS. Failure or overwhelming this anti-oxidant system can result in RBC aging, structure damage, and thus stimulates clearance of those RBCs. Given the high ROS production in COVID-19 which is thought to be generated from neutrophils (26), an RBC clearance due to oxidative stress might explain the low RBCs seen with COVID-19 in some studies.

Several studies have showed that age is related to disease severity and outcome (27, 28). In a meta-analysis study on 36,470 patients from 59 studies, men, and patients over 70 had a higher risk for severe disease, ICU admission, and death (27). Ageing is obviously associated with more co-morbidities, and a decrease in immune and organ function, all of which is expected to increase mortality risk. In our study, age was significantly associated with disease severity and disease outcome, and using a linear regression model, being in the younger age group (18-44) was a more significant factor than albumin levels in predicting a milder disease. Interestingly, unlike other studies gender was not significantly associated with disease severity or disease outcome in our study. Several factors are thought to contribute to sex dependent difference including hormonal, behavioral such as smoking, immunological, and anatomical such as distribution of the angiotensin-converting enzyme 2 (ACE-2) receptors (29). It would be interesting therefore to study these differences in more depth in the Saudi population, and to study whether some specific factors such as the increasing smoking prevalence among the Saudi females, or other genetic factors might mask sex-dependent covid outcome in the Saudi population. However, it is worth noting that a study with a larger sample size would be required to further confirm the results.

Overall, this study aids in discovering predictive factors to COVID-19 severity and outcome in KAUH which was previously deficient, specifically at the time the study was conducted when admission criteria guidelines were set following the initial phase of the disease. However, we acknowledge that the small sample size and being a single center study is a limitation in our study. In addition, the study is a retrospective study which implies that some variables and data that would have given more information to the study were not available, including information that could have classified patients further according to the WHO ordinal severity scale. Furthermore, the lack of control group, for example patients with other respiratory infections, precludes discussion on whether the markers observed are to be increased or decreased in severe COVID-19 are specific to COVID-19 or apply to patients with severe respiratory infections in general.

Laboratory tests are overall, simple, non-invasive, accessible, and can be repeatedly ordered, which makes them attractive for the use in machine learning (ML) models that can contribute in the diagnosis or prognosis of COVID-19. Indeed, despite that early COVID-19 ML models were mainly based on radiographic data, more laboratory based ML models followed after that (30).

Identifying the laboratory markers to be used in diagnostic or prognostic ML models is of crucial importance. For example, markers that are expensive, not routinely available, cannot be ordered fast, or that have a long analytical time would be non-applicable (30). In addition, the markers needs to be of diagnostic value in the relevant population. In a recent systematic review, sixty-eight articles have been identified for using laboratory data only ML models in the diagnosis or prognosis of COVID-19, however, none of those studies were conducted in the Saudi population (30).

We believe that our study aided in predicting parameters that are relevant to the Saudi population and which can together with data from other Saudi studies help build ML models that can enhance patient management.

## Data availability statement

The original contributions presented in the study are included in the article/supplementary material. Further inquiries can be directed to the corresponding author.

## Ethics statement

This study was a retrospective study conducted at KAUH in Jeddah, Saudi Arabia and approved by the Biomedical Research Ethics Committee ethics board at King Abdul-Aziz University (Reference No 360-20).

## Author contributions

The Conceptualization: AA, SE, BE, GA. Data curation: AA, SE. Formal analysis: AA, SE, HB, SA. Writing – review & editing: AA, BE, GA, RB, MB, AE. All authors read and approved the final manuscript. All authors contributed to the article and approved the submitted version.
